# Surgical treatment of aortobronchial fistula after thoracic endograft failure

**DOI:** 10.1186/1749-8090-6-134

**Published:** 2011-10-11

**Authors:** Angelo Maria Dell'Aquila, Stefano Mastrobuoni, Alina Gallo, Isidro Olavide, Alejandro Martin-Trenor

**Affiliations:** 1 Department of Cardiac Surgery, San Martino University Hospital, l.go R. Benzi 10, 16132, Genova, Italy; 2 Department of Cardiovascular Surgery, University of Navarra, Clinica Universitaria, Avenida Pio XII, Pamplona, Spain; 3 Department of Anesthesiology. University of Navarra, Clinica Universitaria, Avenida Pio XII, Pamplona, Spain

**Keywords:** bronchial fistula, aortic dissection, aortic ulcer, endovascular stent

## Abstract

Endovascular stent grafting has been recently considered as a less invasive alternative to either medical therapy or open surgical treatment for many patients with descending thoracic aortic disease. Late complications are rarely described in literature. Herein, we described the occurrence of an aorto-bronchial fistula and a retro-A dissection in a 73-year-old man after stent-grafting for a penetrating atherosclerotic ulcer (PAU) of the descending thoracic aorta and the successful surgical technique adopted in order to remove the stent-graft.

## Background

Endovascular stent grafting has been considered as a less invasive alternative to either medical therapy or open surgical treatment for many patients with descending thoracic aortic disease. However, the Expert Consensus Document on the Treatment of Descending Thoracic Aortic Disease Using Endovascular Stent-Grafts has recently declared that, despite reasonably low early operative morbidity and mortality, late complications of thoracic aortic stent grafting are much more common than those reported for the open aortic surgery [1]. Thus, it is not clear at this time whether the trend toward more aggressive endovascular stent-graft management will affect prognosis, freedom from aortic complications and survival, compared with conventional open surgical repair or medical management alone. To date, late complications described in literature after endovascular stent grafting include endoleaks, graft migration, stent fractures and aneurysm-related death (such as aneurysm rupture and fistulation). Nowadays, the lack of standard surgical protocols and a poor literature raise concerns about how to deal with these complications. Herein, we described a case of aorto-bronchial fistula after endovascular stent implantation and the successful surgical strategy in order to remove the stent.

## Case presentation

A 73-year-old man with a history of smoking and hypertension was admitted to his referring hospital with chest pain and dyspnea. Computed tomography (CT) revealed a penetrating atherosclerotic ulcer (PAU) with intramural hematoma in the distal part of the aortic arch and left hemothorax. Antihypertensive therapy was promptly instituted. A bypass between the left and right carotid arteries was performed and the intimal ulcer was covered by the stent-graft (Zenith Cook 36 mm) in supra-subclavian landing zones; its exclusion was confirmed by the postoperative angiography.

The postoperative course was uneventful and the patient was discharged home on postoperative day 8.

Three months after his discharge, the onset of nausea and hemoptysis required emergent hospitalization.

**Figure 1:**
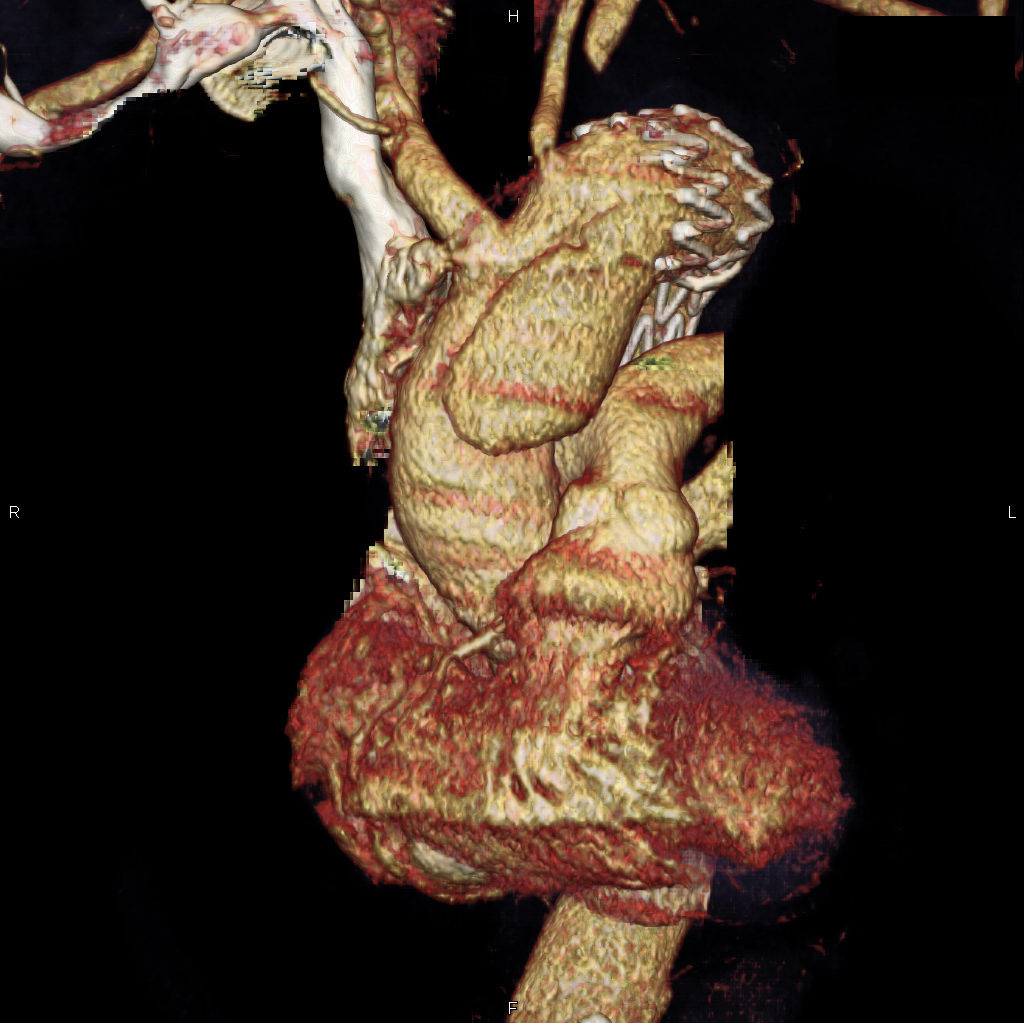
**Three-dimensional computed tomographic reconstruction demonstrating the retro-A dissection**.

**Figure 2:**
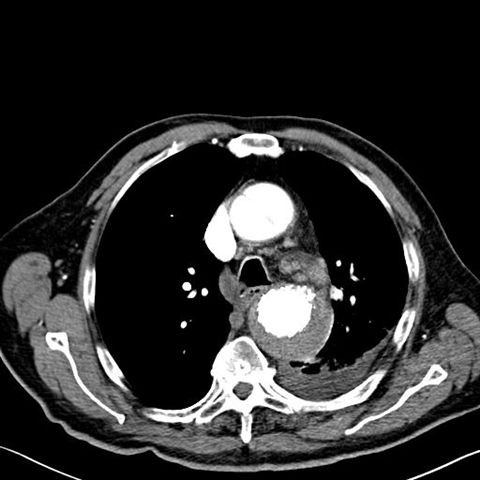
**Computed tomographic showing the periaortic hematoma**.

CT scan showed a retro-A dissection with partially thrombosed false lumen in ascending aorta [Figure 1], extravasation of contrast into perigraft space with a big periaortic hematoma in the area of the distal portion of the stent graft [Figure 2], left apical lung hemorrhage and hemothorax.

The patient was referred to our hospital for an emergent surgical approach.

**Figure 3:**
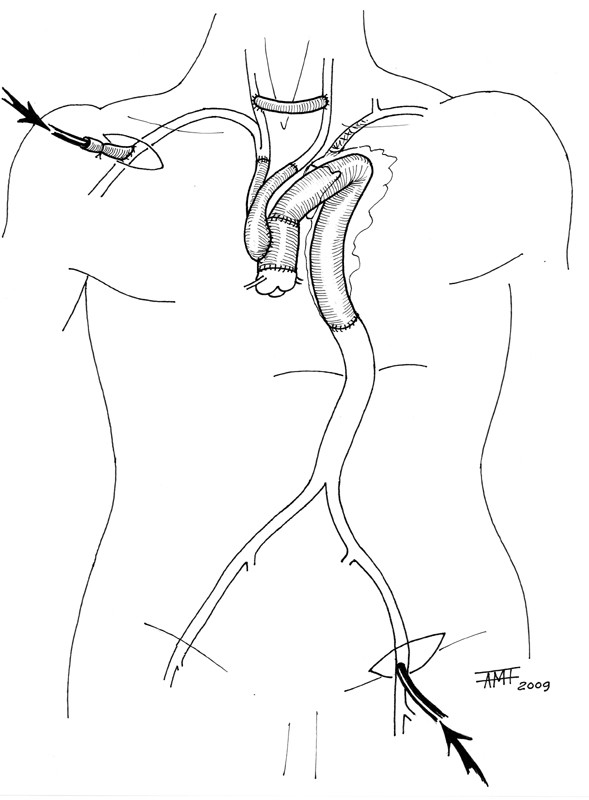
**Picture showing the operative strategy adopted in order to remove the endograft and to replace the ascending aorta, aortic arch, and descending aorta avoiding circulatory arrest**.

The operation was performed with a single-stage approach via bilateral anterior thoracosternotomy. Cardiopulmonary bypass was established using the right axillary artery and right atrium. A clamp was placed on the distal ascending aorta and the ascending aorta was incised. No entry tear was found; the false lumen was partially thrombosed. Cold intermittent blood cardioplegia was delivered antegradely. Once the aortic valve was resuspended and proximal anastomosis was performed with a 30 mm Dacron graft (Hemashield Gold; Boston Scientific Medi-Tech. Wayne, NJ, USA), cooling was initiated in case of circulatory arrest. Once a deep hypothermia (20° C) was reached, brachiocephalic trunk, the left common carotid artery and the descending aorta at level of the diaphragm were clamped and a modified cardiopulmonary bypass was performed starting the flow also through a second femoral artery line. After the left phrenic and left vagus nerves were identified, the aortic arch and the descending aorta were incised and the stent graft was removed. After the completion of the distal anastomosis with a Dacron graft (Hemashield Gold 26 mm), the two grafts were end-to-end sutured. The distal clamp was removed and coronary perfusion was reestablished through the femoral artery line. Perfusate flow was increased and rewarming was initiated. A 20 × 10 mm bifurcated Dacron graft was anastomosed in an end-to-side fashion to the ascending aorta, the brachiocephalic trunk, and the left common carotid artery. Antegrade cardiopulmonary bypass was restarted [Figure 3].

The postoperative period was uneventful excepted for the presence of prolonged pulmonary air leakage. The patient was discharged on postoperative day 35. At 3 month follow up, a contrast-enhanced thoracic CT showed the image of a pseudoaneurysm with a maximum diameter of 75 mm developed at the level of the distal anastomosis. The patient underwent aortic stent grafting (William Cook Europe) without complications.

**Figure 4:**
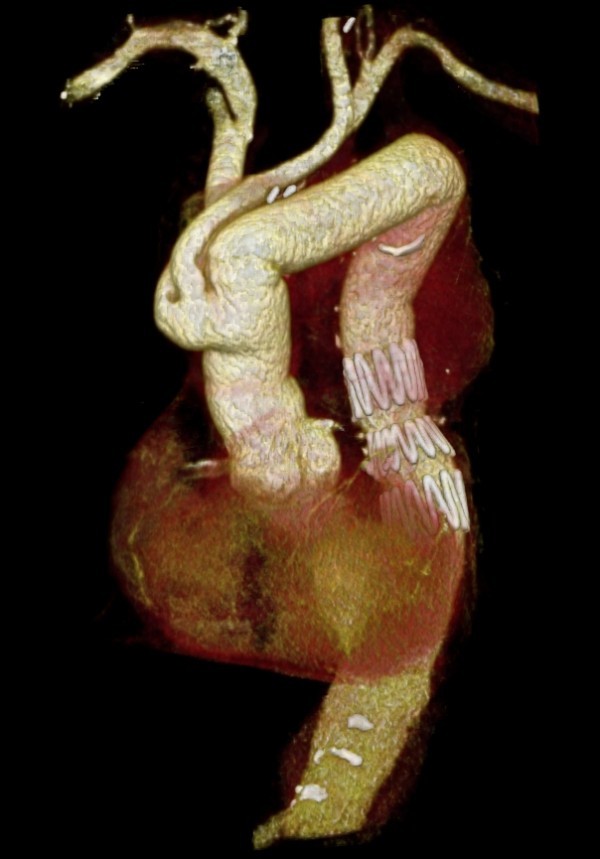
**Three-dimensional computed tomographic reconstruction (2 years follow-up) demonstrating the occlusion of the by-pass between the two carotids**.

At 2 years follow up a CT showed the occlusion of the by-pass between the two carotids [Figure 4]. At this time, the patient was in optimal state of health and no neurological episodes were reported.

## Discussion

Despite recent literature suggesting a significant improvement in outcomes with open surgical repair [2], a less invasive approach for high-risk groups of patients offers the potential for lower morbidity and mortality. Stevenson et al report a significantly lower perioperative mortality and complications rate in the endograft versus the open-surgery control cohort [1]. Although results of endovascular repair are promising, the authors stress the importance of randomized long-term studies also because the use of stent grafts is associated with early and late unique complications that can be difficult to manage [3].

These late complications often require different and difficult approaches that have been partially faced by surgeons using the frozen elephant-trunk via the technique of median sternotomy in deep hypothermia and circulatory arrest or via left thoracotomy using left heart by-pass technique [4-6]. However, in presence of an aorto-esophaegeal or an aorto-bronchial fistula the treatment options are very limited [7,8].

In the present case report, considering the limited mobility of the patient due to knee arthrodesis and the advanced age, a less invasive procedure was chosen as the best alternative to manage the PAU. The stent graft sealed the PAU but two serious complications occurred: an aorto-bronchial fistula and a retro-A dissection. We believe that, because of the poor flexibility of the stent graft, the distal uncovered bare stent eroded the aortic wall causing the intramural hematoma [Figure 2]. The haemoptysis observed three months later was due to the continuous stress produced by the expansive force of the stent against the intimal membrane, resulting in leaking blood into the hematoma and the left main stem bronchus. This hematoma partially limited the loss of blood by covering the leak.

In our case, the bilateral thoracosternotomy provided an optimal exposure of ascending aorta, aortic arch and epiaortic vessels.

The simultaneous cannulation of right axilary and femoral arteries facilitate the sequential clamp of different aorta portions and avoid circulatory arrest maintaining an optimal brain, renal and spinal cord perfusion. Exceptionally, no selective brain perfusion was required thanks to the previous carotid-carotid bypass.

Long-term durability of endografts remains unanswered; we think that patients with endoprosthesis must be strictly followed-up and new standard protocols in management of complications need in order to establish an optimal surgical approach.

## Consent

Written informed consent was obtained from the patient for publication of this case report and accompanying images. A copy of the written consent is available for review by the Editor-in- Chief of this journal.

## Abbreviations

CT: Computed tomography; PAU: Penetrating atherosclerotic ulcer.

## Competing interests

The authors declare that they have no competing interests.

## Authors' contributions

AMD conceived, supervise, literature research, wrote the article. AG participated in its design, writing process and bibliography. AMT, SMT participated in its coordination and correction on the surgical part. IO, SMT;AMT conceived participated in its coordination on the anesthesiologic and extracorporal assistance part. All authors read and approved the final manuscript
